# What lies beyond the eye: the molecular mechanisms regulating tomato fruit weight and shape

**DOI:** 10.3389/fpls.2014.00227

**Published:** 2014-05-27

**Authors:** Esther van der Knaap, Manohar Chakrabarti, Yi Hsuan Chu, Josh P. Clevenger, Eudald Illa-Berenguer, Zejun Huang, Neda Keyhaninejad, Qi Mu, Liang Sun, Yanping Wang, Shan Wu

**Affiliations:** ^1^Department of Horticulture and Crop Science, The Ohio State UniversityWooster, OH, USA; ^2^Department of Pomology, College of Agriculture and Biotechnology, China Agricultural UniversityBeijing, China

**Keywords:** tomato, fruit morphology, gene regulation

## Abstract

Domestication of fruit and vegetables resulted in a huge diversity of shapes and sizes of the produce. Selections that took place over thousands of years of alleles that increased fruit weight and altered shape for specific culinary uses provide a wealth of resources to study the molecular bases of this diversity. Tomato (*Solanum lycopersicum*) evolved from a wild ancestor (*S. pimpinellifolium*) bearing small and round edible fruit. Molecular genetic studies led to the identification of two genes selected for fruit weight: *FW2.2* encoding a member of the Cell Number Regulator family; and *FW3.2* encoding a P450 enzyme and the ortholog of KLUH. Four genes were identified that were selected for fruit shape: *SUN* encoding a member of the IQD family of calmodulin-binding proteins leading to fruit elongation; *OVATE* encoding a member of the OVATE family proteins involved in transcriptional repression leading to fruit elongation; *LC* encoding most likely the ortholog of WUSCHEL controlling meristem size and locule number; *FAS* encoding a member in the YABBY family controlling locule number leading to flat or oxheart shape. For this article, we will provide an overview of the putative function of the known genes, when during floral and fruit development they are hypothesized to act and their potential importance in regulating morphological diversity in other fruit and vegetable crops.

## Introduction

**Figure 1:**
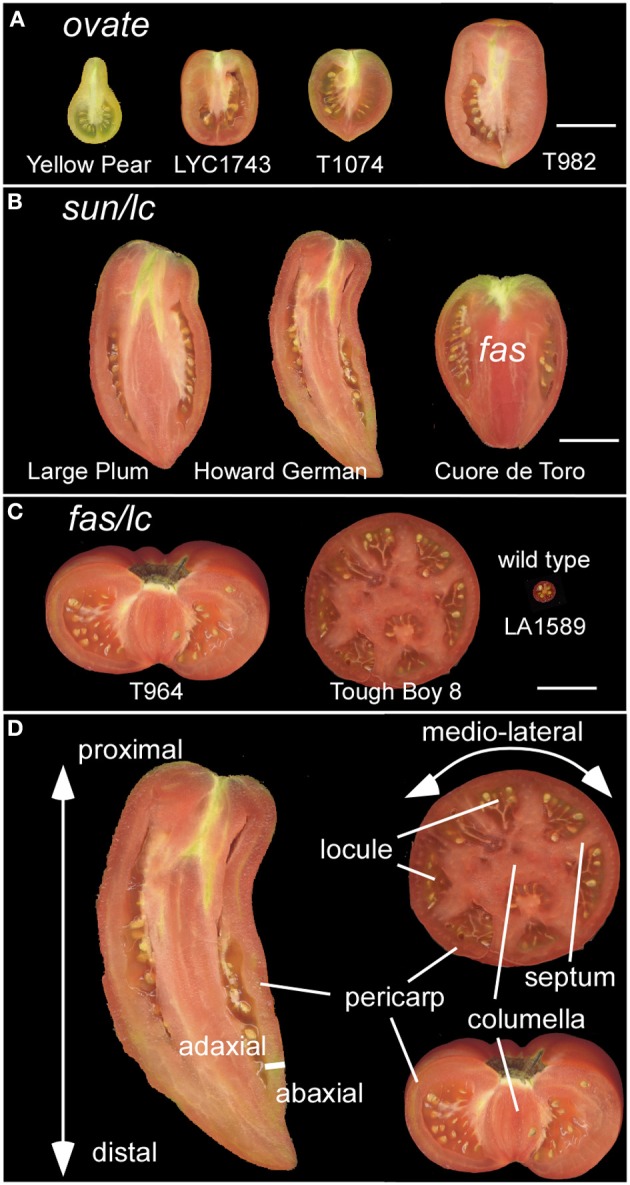
**Diversity in tomato fruit shapes**. **(A)** Tomato varieties carrying the *ovate* mutation result in obovoid and ellipsoid fruit shapes. Size bar = 3 cm. **(B)** Tomato varieties carrying the *sun* and *lc* mutation result in a long fruit shape. The oxheart shaped tomato also carries *fas* in addition to *sun* and *lc*. Size bar = 3 cm. **(C)** Tomato varieties carrying *fas* and *lc* result in a flat fruit with many locules. The wild type represents the fruit from an ancestor of cultivated tomato, *S. pimpinellifolium* LA1589. Size bar = 3 cm. **(D)** The axes of growth: proximal (closest to the stem) to distal (farthest away from the stem); medio-lateral; and the adaxial (closest to the meristem) to abaxial (farthest from the meristem). The fruit tissues that give structure to the organ are highlighted as the pericarp, septum, columella and locule. Not indicated is the placenta which is the tissue extending from the columella and surrounds the seeds.

Angiosperm plants vary tremendously in morphological traits related to their reproduction. The floral appearance is driven by evolutionary aspects of the pollination syndrome whereas distinct dispersal modes drive the evolution of phenotypes associated with the fruit. In natural settings, the main functions of the fruit are to protect the developing seeds and to act as a dispersal agent. The onset of the change to an agricultural lifestyle, approximately 10,000 years ago, provided strong selection pressures on the fruit of incipient vegetable and fruit crops. The selections made by early farmers offer a great opportunity to identify the molecular basis of a range of phenotypic traits, especially those related to fruit morphology and flavor. For example, selections against bitter taste resulted in palatable eggplant and cucumber (Wang et al., 2008; Qi et al., 2013). Yet, the underlying principle for nearly all cultivated vegetable and fruit crops was the selection for larger and more nutritious fruits featuring a variety of shapes (Paran and Van Der Knaap, 2007; Pickersgill, 2007; Meyer and Purugganan, 2013) (Figures 1A–C). The larger fruit became more nutritious as a result of the increase in the edible and fleshy part of the fruit at the expense of the seed part for most domesticated fruits and vegetables.

The focus of the “hypothesis and theory” article is to summarize the current knowledge on the function of genes that change tomato fruit weight and shape resulting from domestication and diversification process. The focus on tomato is based on the extensive research that resulted in the cloning of six fruit shape and weight genes from this species in recent years. The predicted function of these genes will be discussed in the context of the phases of development where we hypothesize the impact of the mutant alleles is most critical. It is important to recognize that the mutations are not often resulting in complete nulls, i.e., a loss-of-function allele. Thus, the complete repertoire of functions of the tomato fruit shape and weight genes may not be apparent from the phenotype observed in the natural mutants. We will propose the pathways in which the shape and weight proteins function. We will also include the molecular basis of the underlying mutations that gave rise to the derived alleles and demonstrate that inversions, duplications, as well as single nucleotide polymorphisms (SNPs) in promoters and coding regions underlie the phenotypic diversity of the tomato fruit.

## Overview of tomato development

**Figure 2:**
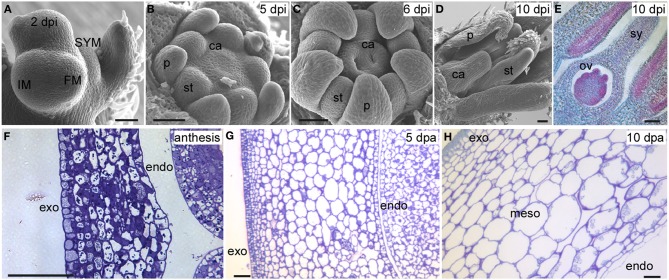
**Tomato floral and fruit development in *S. pimpinellifolium* LA1589**. **(A)** The IM terminates into the FM, which develops into a floral bud 2 dpi. The SYM is emerging from the flank of the youngest leaf primordium. **(B)** Emergence of carpel primordia 5 dpi. **(C)** Growth of carpels and formation of two locules 6 dpi. **(D)** Growth of the style. **(E)** Formation of the ovules 10 dpi. **(F)** A transverse section of the pericarp at anthesis. **(G)** Transverse section of the pericarp 5 dpa. **(H)** Transverse section of the pericarp 10 dpa. SYM, sympodial meristem; IM, inflorescence meristem; FM, floral meristem; dpi, days post floral initiation; dpa, days post anthesis; p, petal; st, stamen; ca, carpel; sy, style; ov, ovule; exo, exocarp; endo, endocarp; meso, mesocarp. Size bar is 50 μm.

Even though the fruit is a terminal structure that forms relatively late in the plant's lifecycle, the formation of this organ and the parameters that determine its final dimensions are rooted much earlier in the plant's lifespan. Therefore, it is important to view tomato fruit development in the context of overall plant development starting after germination. Plant growth in tomato and other Solanaceous plants is characterized by a sympodial shoot architecture where after formation of 8–10 leaves, the shoot apical meristem (SAM) terminates into the inflorescence meristem (IM), and growth continues from lateral meristems called sympodial meristems (SYM). Meanwhile, the IM terminates into the floral meristem (FM) generating the flower (Schmitz and Theres, 1999). The tomato inflorescence also features a sympodial structure since a new IM emerges simultaneously from the flank of the first FM, terminating again in the second FM on the inflorescence and so on (Figure 2A). This growth pattern is referred to as cymose and results in a zigzag of flowers on a tomato inflorescence (Welty et al., 2007; Lippman et al., 2008; Castel et al., 2010). In most angiosperm species, FMs give rise to four whorls: the sepals, petals, stamens and carpels. Organ identity genes play critical roles to ensure that carpel primordia arise from specified founder cells within the FM (Causier et al., 2010) (Figures 2A,B). In addition to cell specification, the establishment of the boundaries between and within the primordia is required to ensure that the appropriate identities and division patterns are initiated and maintained throughout gynoecium growth (Dinneny and Yanofsky, 2005; Balanza et al., 2006; Girin et al., 2009). This step is critical to lay the foundation of growth of the organs along three axes: the proximal-distal, the medio-lateral and the abaxial-adaxial axis (Figure 1D). A mature tomato gynoecium coincides with flower opening which marks the anthesis and pollen release stage (Xiao et al., 2009). Following pollination and fertilization of the ovules, fruit development is initiated which is marked by a rapid increase in cell proliferation followed by cell enlargement (Gillaspy et al., 1993; Xiao et al., 2009) (Figures 2G,H). In most fruit tissues such as the pericarp, cell division ceases 5–10 days after anthesis and growth of the fruit continues by extensive cell enlargements that last for three to 5 weeks until the fruit ripening stage (Gillaspy et al., 1993; Xiao et al., 2009).

## Critical developmental stages before anthesis at which the final shape and weight of fruit is regulated

The final dimensions of the fruit are regulated during multiple stages throughout the development of the plant. These stages occur before and after anthesis, and may be initiated as early as in the SAM. Thus, the first stage of regulation of the final fruit dimensions is likely to occur in the meristems as a result of their size (Figure 2A; Table 1). Since the gynoecium is a terminal structure, the size of the FM may impact the number of cells that are specified to form a carpel primordium as well as the number of primordia (Szymkowiak and Sussex, 1992; Clark et al., 1993; Taguchi-Shiobara et al., 2001; Suzaki et al., 2004). Cell identity and the positioning of organ primordia *per se* however are not controlled by the size of the FM. Therefore, the second stage of regulation is likely controlled by the organization within the meristem which relates to where and how often in the meristem the cells that are destined to become carpel primordia arise (Figures 2A,B). Similarly as for leaf primordia initiation, localized auxin maxima controlled by the auxin efflux protein PIN1 and the expression of PLETHORA/AINTEGUMENTA transcription factors are thought to control floral organ positioning (Benkova et al., 2003; Krizek, 2011; Van Mourik et al., 2012; Hofhuis et al., 2013). The areas of low auxin coincide with the boundaries between primordia which are also tightly controlled processes (Nahar et al., 2012; Zadnikova and Simon, 2014). Misalignment during this stage would result in changes in final fruit morphology. The third stage is the phase that transmits positioning information to gynoecium growth (Figures 2B,C, Table 1). During this phase, the three axes of growth have been specified along which cell proliferation and enlargement occurs (Dinneny et al., 2005; Ostergaard, 2009). Cell proliferation, which is characteristic of this stage, consists of the rate and duration of the cell divisions within the developing ovary impacting final organ dimensions (Figure 2C). Also critical are the differential rates and duration of cell division within distinct tissues in the developing ovary, resulting in alternatively shaped fruit. For example, ovary and fruit length is determined by the degree of growth along the proximal-distal axis whereas width is determined by the degree of growth in the medio-lateral axis (Figure 1D). The degree of the pericarp thickness and other internal tissues is determined along the abaxial-adaxial axis. Therefore, enhanced cell divisions preferentially along one axis of growth are proposed to lead to a different shape fruit as opposed to enhanced cell divisions along all three axes of growth. The fourth stage occurs concomitantly with the third stage which is the continued specification of new tissue types through reactivation of the meristematic potential leading to the formation of many tissue types (Girin et al., 2009) (Figures 2D,E). Along the proximal-distal axis, the gynoecium develops two additional regions: the stigma and style. Along the medio-lateral axis, the ovary develops the placenta, ovules and transmitting track tissues. Along the abaxial-adaxial axis the ovary continues to maintain the polarities within the different tissues such as the pericarp, septum, placenta and ovules. The reinforcement to maintain the different zones is mediated by transcription factors in conjunction with boundary genes (Heisler et al., 2001; Nahar et al., 2012).

**Table 1:** **Developmental phases proposed to control fruit shape and weight**.

**Critical regulatory phases of fruit shape and weight**	**Developmental event^1^**	**Landmark^1^**	**Cellular events in the ovary or fruit**	**Days after meristem initiation**	**Stage-specific fruit shape and weight genes**
Phase 1	Inflorescence and floral meristem formation	Floral landmark 1	Cell number, size of the stem cell niche	0	*LC/FAS/CNR*
Phase 2	Floral meristem organization	Floral landmark 1	Cell identity and boundary information	1	*FAS/CNR*
Phase 3	Gynoecium initiation	Floral landmark 5	Cell proliferation and enlargement	5–6	*OVATE/SUN*
Phase 4	Gynoecium growth	Floral landmark 6–9	Rediffentiation of tissue types	8–16	
Phase 5	Anthesis	Floral landmark 10 and fruit landmark 1	Flower opening	19	
Phase 6	Fertilization and 4–16 cell stage embryo	Fruit landmark 2–3	Cell proliferation	20–25	*SUN/KLUH*
Phase 7	Globular to coiled stage embryo	Fruit landmark 4–7	Cell enlargement	25–39	

1 From Xiao et al. (2009).

## Critical developmental stages after anthesis at which the final shape and weight of fruit is regulated

The anthesis/pollination/fertilization phase marks the end of ovary development and the beginning of fruit development. Lack of or poor fertilization leads to changes in fruit shape and reduced weight, marking the fifth phase. Aborted fruit is terminal and should not been considered to be part of phase 5. The first stage post-anthesis is the sixth phase proposed to correspond to the cell proliferation stage, a rapid increase in cell division throughout the developing fruit that follows immediately after fertilization (Gillaspy et al., 1993; Xiao et al., 2009) (Figures 2F,G). As in the ovary, this stage is comprised of differing cell division rates and duration in the tissues of the fruit that would greatly impact final fruit shape. The seventh and final stage is proposed to be cell enlargement which impacts overall fruit size the most (Figure 2H). Cell enlargement is regulated differentially in the various tissues within the fruit, and rates and duration determine the final fruit dimensions. For example, the columella and placenta tissues contain more large cells than the pericarp. Additionally within the pericarp, the exocarp cells (constituting the epidermis) are very small whereas the mesocarp cells are large (Figure 2H).

## Tomato fruit weight and shape alleles acting pre-anthesis

### LOCULE NUMBER

**Figure 3:**
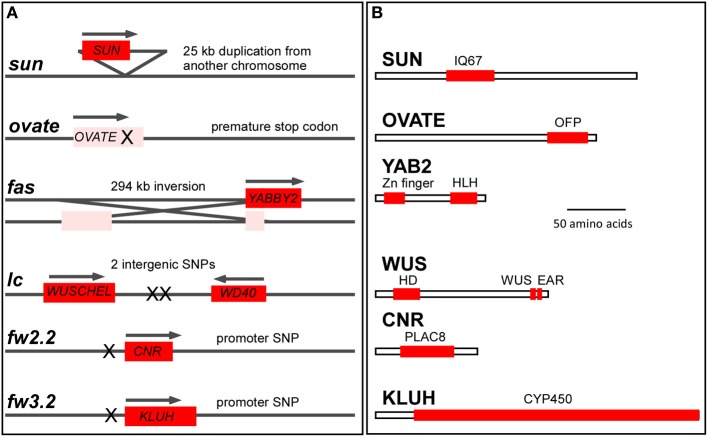
**The molecular basis of tomato fruit shape and weight variation**. **(A)** Genome structure of the fruit shape and weight loci and the underlying mutations. Red box indicates the coding region of a functional gene whose regulation is altered by the mutation (denoted by X). Pink indicates a loss-of-function mutation of the gene. The size of the loci are not drawn to scale. **(B)** Protein features of the fruit shape and weight proteins. The box represents the coding region. The most important domains are listed as red boxes. IQ67, CaM binding domain of 67 amino acid and containing IQ; OFP, Ovate Family Protein motif of unknown function; HLH, YABBY type of DNA binding domain featuring a helix-loop-helix structure; HD, DNA binding homeodomain of the helix-loop-helix-turn-helix structure; WUS, essential for proper functioning of WUSCHEL; EAR, transcriptional repressor function; PLAC8, similarity to the placenta-specific gene 8 protein; CYP450, cytochrome P450. Size bar = 50 amino acids.

**Figure 4:**
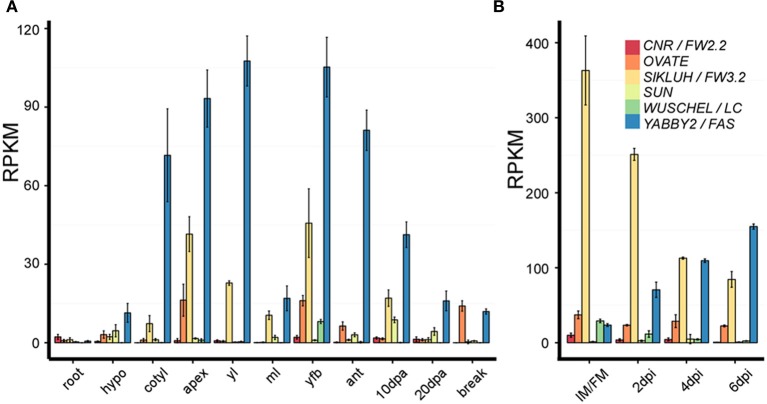
**Expression analysis of the six fruit shape and weight genes in different tissues and at different developmental stages**. Samples were collected from the *S. pimpinellifolium* accession LA1589. The data was obtained from 3 to 4 biological replicate RNA samples that were sequenced using the HiSeq2000 Illumina sequencing technology (Huang et al., 2013) (http://ted.bti.cornell.edu/cgi-bin/TFGD/digital/home.cgi). The expression was normalized using the reads per kilobase per million mapped reads of each gene model (RPKM). **(A**) The root, hypocotyl (hypo), cotyledon (cotyl) and shoot apex including the SAM (apex) were collected from the same seedlings germinated in petri dishes. All other tissues were collected from mature plants grown in the greenhouse (Huang et al., 2013). yl, young leaves; ml, mature leaves; yfb, young floral buds from 10 dpi and younger; ant, whole flower at anthesis; 10 and 20 dpa, developing fruit 10–20 days after anthesis; break, breaker stage fruit which is immediately before turning color. **(B)** IM/FM, 2, 4, 6 dpi flower buds that were fixed in RNAlater solution. The tissues were hand-dissected using a dissecting scope prior to RNA isolation. Each replicate out of 3 is represented by 100–150 samples that were pooled prior to RNA extraction.

*LOCULE NUMBER* (*LC*) controls the number of carpel primordia and a mutation results in a fruit with more than the typical two to three locules (Barrero et al., 2006; Munos et al., 2011). Increases in locule number often lead to a flat fruit of a larger size and the mutation is common in beefsteak tomato and tomatoes on the vine (Munos et al., 2011; Rodriguez et al., 2011) (Figure 1C). Since carpel primordia arise early in floral development, it is likely this gene functions in regulating meristem size and/or in the initiation of organ primordia. The locus was fine mapped to a 1608 bp region located between a putative ortholog of *WUSCHEL* (*WUS*) (annotated gene ID Solyc02g083950, available at http://solgenomics.net/) and a WD40 motif containing protein (Solyc02g083940). Further association mapping led to the identification of two single nucleotide polymorphisms located 1080 bp downstream of the putative tomato ortholog of *WUS* (Munos et al., 2011) (Figure 3). *WUS* encodes a homeodomain transcription factor that is required for maintaining the stem cell identity in the SAM (Mayer et al., 1998; Clark, 2001). The WD40 containing motif protein belongs to a large family involving in diverse functions ranging from signal transduction to transcriptional regulation (Ullah et al., 2008). Increased expression of *WUS* in Arabidopsis leads to increased floral organ number, which is similar to the phenotype found in the *lc* mutant (Mayer et al., 1998; Clark, 2001). Therefore, based on the predicted function *SlWUS* is the most likely candidate to underlie *lc*, impacting the first phase that regulates the final dimension of the tomato fruit (Figure 2A and Tables 1, 2). Similar to Arabidopsis, *SlWUS* is expressed in the youngest floral buds and the shoot apex and virtually undetectable in other tomato tissues (Figure 4A). Its expression is also high in the IM/FM tissues, decreasing very rapidly as floral development progresses (Figure 4B).

**Table 2:** **List of genes controlling fruit weight and shape variation in tomato**.

**Locus/QTL**	**Underlying gene ID**	**Putative cellular/molecular function and length of the protein**	**Timing of the impact on morphology**	**Most likely cause of allelic variation**	**References**
*fw2.2*	*Cell number regulator (CNR)* Solyc02g090730	Increased expression is associated with reduced cell division. May permit transport across membranes. Protein may be located at the plasmamembrane and contains a PLAC8 domain including two putative transmembrane motifs. 163 aa	Phase 1 or 2 (Figures 2A,B)	SNP in the promoter of the gene	Frary et al., 2000; Guo et al., 2010
*fw3.2*	*KLUH* Solyc03g114940	A cytochrome P450 of the 78A class and the likely ortholog of *AtKLUH*. Hypothesized to synthesize a mobile signal. Substrate unknown. 516 aa	Phase 5, (Figure 2G)	SNP in the promoter of the gene	Anastasiou et al., 2007; Chakrabarti et al., 2013
*lc*	*WUSCHEL* Solyc02g083950	Homeobox domain protein. Required to maintain stem cell identity in meristems. 73 aa	Phase 1, (Figure 2A)	Two SNP located downstream of *WUSCHEL*	Mayer et al., 1998; Munos et al., 2011
*fasciated*	*YABBY2* Solyc11g071810	Transcription factor involved in organ polarity and meristem organization. 177 aa	Phase 1 or 2, (Figures 2A,B)	Gene knock out by a 294 kb inversion with a breakpoint in the first intron of *YAB2*	Cong et al., 2008; Huang and Van Der Knaap, 2011; Huang et al., 2013
*ovate*	*OVATE* Solyc02g085500	Increased expression is associated with shorter plants and plant organs. May be a repressor of transcription. Contains the OFP domain. 352 aa	Phase 3, (Figures 2B,C)	Premature stop codon in an exon associated with a mutant phenotype	Liu et al., 2002; Hackbusch et al., 2005; Huang et al., 2013
*sun*	*SUN* Solyc10g079240	Increased expression is associated with elongated fruit. Positive regulator of growth. Contains the IQ67 motif that binds calmodulin. 421 aa	Phase 3 and 6, (Figures 2B,C,G)	Interchromosomal gene duplication mediated by the transposon *Rider*	Abel et al., 2005; Xiao et al., 2008; Huang et al., 2013

**Figure 5:**
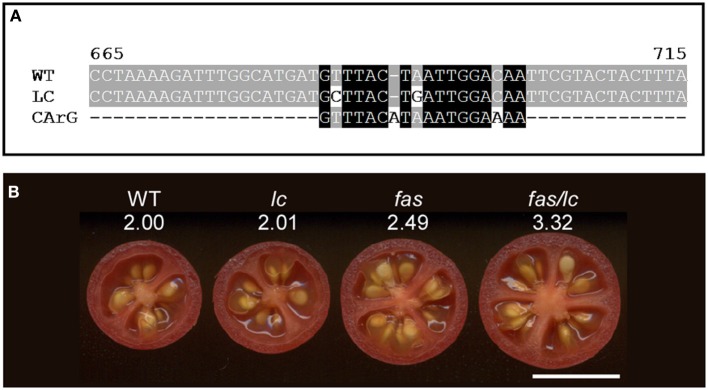
**The effect of *lc* and *fas* loci on locule number in tomato**. **(A)** Alignment of the wild type (accession JF284938) and mutant (JF284939) *LC* allele sequences with the canonical MADS box transcription factor CArG1 binding sequence (Tilly et al., 1998). The two mutations in *LC* reduce the alignment to the consensus sequence. **(B)** The effect on locule number in the *fas*, *lc* and the double *fas/lc* NIL compared to wild type (WT). The number below the NIL indicates the average locule number from over 40 fruit evaluated each from 5 plants. The increase in locule number in the double NIL indicates synergistic interactions of the two mutations. Size bar = 1 cm.

*WUS* is critical in the regulation of the stem cell population size in all meristems, yet the *lc* mutation itself does not lead to dramatic changes in *SlWUS* gene expression compared to wild type (Munos et al., 2011). Therefore, the high locule number phenotype is likely due to subtle changes in expression that were not captured by the method of gene expression quantification. WUS positively regulates the expression of the MADS box transcription factor *AGAMOUS* (*AG*) (Lenhard et al., 2001; Lohmann et al., 2001) and AG is critical in determining stamen and gynoecium identity (Yanofsky et al., 1990). Therefore, WUS-induced expression of *AG* links meristem activities to organ identity processes. AG in turn down-regulates expression of *WUS* providing the mechanism for changing stem cell identity of the remaining FM to carpel identity (Lohmann et al., 2001; Liu et al., 2011). In Arabidopsis, *WUS* down-regulation is mediated by two downstream CArG cis-regulatory elements to which AG binds, resulting in the epigenetic silencing of *WUS* (Tilly et al., 1998; Liu et al., 2011). Intriguingly, the two SNPs located downstream of tomato *WUSCHEL* are located in a putative tomato CArG cis-regulatory element (Figure 5A). This suggests that the *lc* mutation causes a loss-of-function regulatory element permitting higher expression of *SlWUS* and maintenance of a larger stem cell population resulting in increased locule numbers. Furthermore, this finding implies that the *lc* mutation acts at the transition from stem cell identity to carpel identity acting just prior to the stage shown in Figure 2B. Other critical components of the WUS signaling pathway are provided by the CLAVATA (CLV) proteins (Clark, 2001; Brand et al., 2002; Lenhard and Laux, 2003). In particular, the WUS and CLV3 feedback loop is tightly linked to the regulation of meristem size in Arabidopsis (Schoof et al., 2000), suggesting that members of the CLV pathway may be involved in the regulation of tomato meristem size and its organization leading to changes in locule number and the final shape of the fruit.

### Fasciated

The mutation in *FASCIATED* (*f* or *fas*) leads to increases in locule number with more pronounced effects on locule number than *lc* (Lippman and Tanksley, 2001). *fas* is found in certain heirloom tomatoes and a few commercially grown beefsteak varieties (Rodriguez et al., 2011) (Figures 1B,C). In addition to increased locule number, the *fas* mutation results in increased number of all floral organs (Lippman and Tanksley, 2001; Barrero and Tanksley, 2004). Significant epistatic interactions have been detected between *lc* and *fas* (Lippman and Tanksley, 2001; Barrero and Tanksley, 2004), suggesting that both genes act together by co-regulating a core pathway that controls locule number. *FAS* was fine mapped to the bottom of chromosome 11 and, contrary to previously reported results, the mutation resulted from a 294 kb inversion with one of the breakpoints in the first intron of a member of the *YABBY* family creating a null mutation (Huang and Van Der Knaap, 2011). This *YABBY* member, *SlYABBY2* (*YAB2*) is considered to underlie *fas* (Cong et al., 2008) (Figure 3). Compared to any other fruit shape or weight gene, *YAB2* expression is very high in cotyledons, shoot apex, young leaves, young floral buds, and anthesis stage flowers (Figure 4A). In IM/FM and developing floral buds, its expression is relatively low in the meristem but increases in flower buds 6 days after initiation (Figure 4B).

The *YABBY* family of transcription factors is known to control the abaxial-adaxial polarity of SAM, IM, and FM, while also specifying the cell fate of the abaxial region in lateral organs. YABBY proteins function redundantly with other polarity proteins and are required to establish the proper boundaries within the meristem and developing organ primordia (Bowman and Smyth, 1999; Bowman et al., 2002). Moreover, YABBYs have been shown to impact the signaling from lateral organs to the meristem and coordinately maintain the normal growth of meristem in Arabidopsis and rice (Goldshmidt et al., 2008; Tanaka et al., 2012). Because of the function of YABBY family proteins and its expression pattern, we consider that *FAS* is controlling the second stage of final fruit size and shape regulation by impacting meristem organization and boundary information (Figure 2B, Table 2). However, because of its epistatic interaction with *LC*, it is also possible that *FAS* impacts meristem size as well as organization (Figure 2A). The details of how YAB2 impacts locule number are not well understood.

Of the two loci controlling locule number, *lc* and *fas*, the former mutation is much more widespread in the tomato germplasm than the latter while the latter has a more dramatic effect on locule number resulting in up to countless locules per fruit (Munos et al., 2011) (Figures 1B,C). In near-isogenic lines (NILs) using the wild species LA1589 as the background, the impact of these two genes on locule number is much less dramatic (Figure 5B), supporting the notion that in the cultivated background modifiers of these mutations exist. Further genetic analyses would reveal the molecular nature of those modifiers. The epistatic interaction between the two loci is clearly evident in the wild species background as locule number increase in the double NIL is higher than the sum of locule number found in the single NILs (Figure 5B).

### Ovate

**Figure 6:**
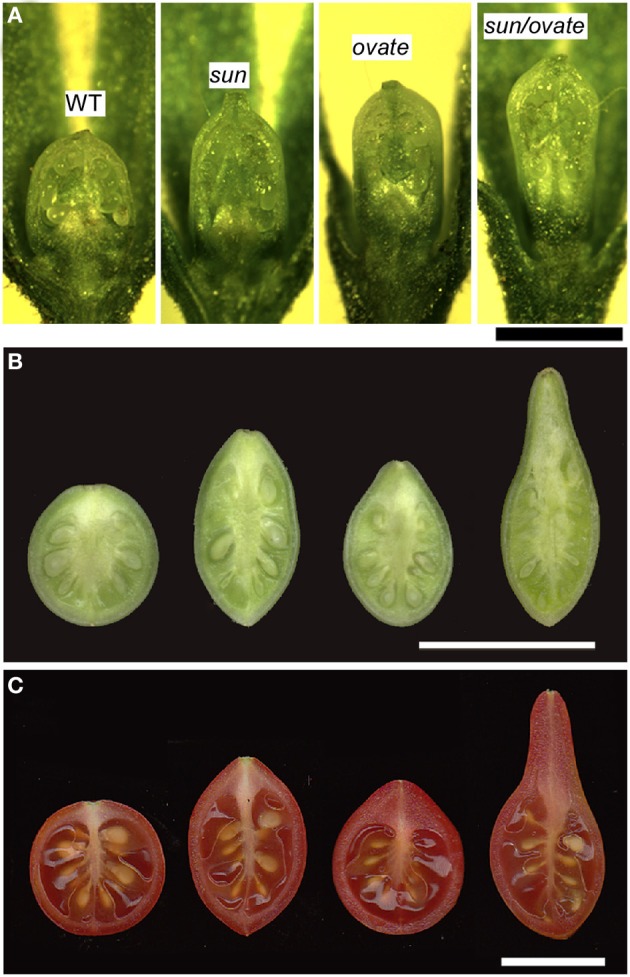
**The effect of the *sun* and *ovate* loci on fruit elongation. (A)** Effect of wild type (WT), *sun*, *ovate* and *sun/ovate* on ovary shape at anthesis. Size bar = 1 mm. **(B)** Effect of WT, *sun*, *ovate*, and *sun/ovate* on fruit shape 10 days post anthesis. **(C)** Effect of WT, *sun*, *ovate* and *sun/ovate* on mature fruit shape. The shape in the double NIL indicates synergistic interactions of the two mutations. Size bar in B and C = 1 cm.

The shape of many ellipsoid and obovoid varieties such as those found in grape tomato is controlled by the gene that regulates fruit elongation, *OVATE* (Ku et al., 1999; Liu et al., 2002; Rodriguez et al., 2011) (Figure 1A). The gene was fine mapped to chromosome 2 and the mutation resulted in a premature stop codon in a newly defined class of plant proteins, Ovate Family Proteins (OFP) (Liu et al., 2002; Hackbusch et al., 2005) (Figure 3 and Table 2). The expression of wild type OVATE is the highest in the shoot apex, youngest floral buds and breaker stage fruit (Figure 4A). Additionally, even though *OVATE* expression is the highest in the IM/FM, expression is reduced by only ~30% in flower buds 2, 4, and 6 days after initiation (Figure 4B); the latter stage corresponds to the stage shown in Figure 2C. Not all tomato varieties that carry the *ovate* mutation display an elongated shape which led to the mapping of two suppressor loci, *sov1* and *sov2*, on chromosomes 10 and 11, respectively (Rodriguez et al., 2013). These suppressors are thought to play important roles in the regulation of shape mediated by the OVATE pathway. OVATE does neither affect floral organ identity, FM organization nor floral organ number (Liu et al., 2002). Instead, OVATE appears to have a specific role in the regulation of anisotropic growth along the proximal-distal axis at the proximal end of the fruit (Figure 6). Near-isogenic lines carrying the *ovate* mutation show that shape is already determined at anthesis (Van Der Knaap and Tanksley, 2001) (Figure 6A) and obovoid shape gradually decreases during the development of the fruit (Figures 6B,C).

**Figure 7:**
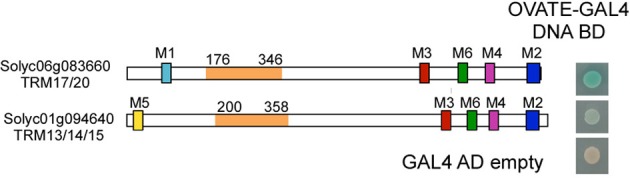
**Protein structure of two TONNEAU1 Recruiting Motif (TRM) proteins that interact with tomato OVATE in a Y2H study. Full length OVATE was used as bait and a truncated form of tomato TRM proteins were used as prey**. The clones were grown on medium selecting for the bait and prey plasmids whereas the X-α-Gal staining highlights the strength of the interaction. The most likely Arabidopsis ortholog is listed below the gene annotation number. Solyc06g083360 interacts strongly and Solyc01g094640 interacts weakly with OVATE. The colored boxes designate the M1-M6 motifs defining the TRM family (Drevensek et al., 2012). The orange domain in the protein is the overlapping region of different prey fragments identified in the Y2H screen and the numbers indicate amino acid positions.

The molecular function of OVATE and its family members are not well understood. Yeast two Hybrid (Y2H) screens using Arabidopsis KNOX and BELL transcription factors as bait led to the identification of OFP members, lending support for the notion that OVATE interacts with patterning genes that impact fruit shape at the early stages of gynoecium development (Hackbusch et al., 2005; Wang et al., 2010). OFP members have also been shown to repress transcription (Wang et al., 2007, 2011) and overexpression of *AtOFP1* leads to dwarf phenotypes in Arabidopsis and tobacco, in part by negatively regulating the transcription of *GA20ox1*, a key gene in the gibberellin biosynthesis pathway (Hackbusch et al., 2005; Wang et al., 2007). Contrary to findings in Arabidopsis, Y2H of the tomato OVATE protein as bait did not lead to the identification of transcription factors including KNOX or BELL. Instead, 11 out of 26 members of the TONNEAU1 Recruiting Motif (TRM) superfamily were identified including the putative ortholog of AtTRM17/20 (Figure 7 and Table 3). Of all interacting clones obtained, 63.8% belonged to the TRM family. The TRM clones identified from the screen were partial clones and the overlap between interacting clones of the same gene is highlighted in orange (Figure 7). TRMs interact with TONNEAU1a (TON1a), TON1b and TON2/FASS proteins, which play critical roles in preprophase band formation and microtubule array organization (Camilleri et al., 2002; Azimzadeh et al., 2008; Spinner et al., 2010, 2013; Drevensek et al., 2012). This finding suggests that OFPs interact with TRMs and microtubules in addition to acting as transcriptional repressors, and thus could provide a mechanistic link between organ patterning and growth. TON1a, TON1b and TON2 interact with the TRM via the M2 and M3 motifs, respectively whereas the TRM motif that recognizes OVATE has not yet been identified. Most single knockouts of Arabidopsis *OFP*s exhibit no or mild phenotypes (Pagnussat et al., 2007; Li et al., 2011; Wang et al., 2011). On the contrary, the premature stop codon mutation found in tomato *OVATE* causes a dramatic morphological change in ovary shape, suggesting it may be a unique member of the family. These findings together suggest that OVATE acts early in carpel development, possibly during phase 3 corresponding to the link between primordia initiation and positioning to growth of the developing carpels.

**Table 3:** **Tomato TONNEAU1 Recruiting Motif proteins (TRM) that interact with OVATE in the Y2H screen**.

**Tomato Gene ID**	**Arabidopsis Ortholog^1^**	**PBS^2^**	**Number of clones**	**Percentage of total**
Solyc07g008670.2.1	TRM5	A	31	16.8
Solyc09g005750.2.1	TRM19	A	27	14.6
Solyc06g083660.2.1	TRM17/20	A	16	8.7
Solyc03g115000.2.1	TRM3/4	A	8	4.3
Solyc02g082680.2.1	TRM26	A	8	4.3
Solyc09g063080.1.1	TRM17/20	B	7	3.8
Solyc01g094640.2.1	TRM13/14/15/33	B	7	3.8
Solyc07g032710.2.1	TRM30/34	B	5	2.7
Solyc03g006840.2.1	TRM25	C	6	3.2
Solyc08g081160.2.1	TRM13/14/15/33	C	2	1.1
Solyc12g007140.1.1	TRM30/34	D	1	0.5

1 The most likely ortholog(s) in Arabidopsis were determined based on BLAST search against TAIR10 Arabidopsis proteins.

2 PBS, Predicted Biological Score, which is computed to assess the reliability of the interaction. A denotes strong and reliable interaction and D denotes weak and/or questionable interaction.

### SUN

*SUN* controls fruit elongation, including those found in commercially grown plum tomatoes, the very long and tapered shaped heirloom and oxheart tomatoes (Rodriguez et al., 2011) (Figure 1B). *SUN's* effect on fruit elongation is much more pronounced than the effect of *OVATE* (Figures 1A,B, 6). The locus was fine mapped to the short arm of chromosome 7 and found to encode a member of the IQD family of calmodulin-binding proteins (Van Der Knaap et al., 2004; Xiao et al., 2008). The mutation arose from a highly unusual 24.7 kb duplication event from chromosome 10 to chromosome 7 (Jiang et al., 2009) (Figure 3, Table 2). This transposition was mediated by the retrotransposon *Rider*, which has also been found to underlie mutations at a few other loci in cultivated tomato unrelated to fruit shape (Jiang et al., 2012). Expression of wild type *SUN* is found in 10 days post anthesis fruit but in general is extremely low in all tissues examined (Figure 4). The duplication placed *SUN* in a new genome environment leading to much higher expression throughout floral and fruit development and an extremely elongated fruit (Xiao et al., 2008, 2009).

The effect of *SUN* on fruit shape is noticeable at anthesis albeit that the effect of the gene is more pronounced immediately following fertilization (Van Der Knaap and Tanksley, 2001; Xiao et al., 2009; Wu et al., 2011) (Figures 6A,B). The results suggest that *SUN* sets up the patterning before anthesis during gynoecium development whereas the execution of the patterning plan occurs in part after fertilization. Interestingly, *SUN* also controls sepal and terminal leaflet shape and high expression leads to twisted stems and leaf rachises (Wu et al., 2011) implying a role for this gene in lateral (leaf and sepal) as well as terminal (fruit) organ development. Epistatic interaction of *SUN* and *OVATE* is likely with respect to growth of the proximal part of the fruit (Figure 6C). The degree of obovoid (pear) shape is much pronounced in the double NIL than in the sum of the single NILs.

SUN changes fruit shape by redistributing fruit mass; an increase in cells in the proximal-distal direction is accompanied by a decrease in cell number in the columella and septum in the medio-lateral direction throughout the entire fruit (Wu et al., 2011) (Figures 6A–C). This suggests that alterations in cell division patterns are critical for fruit shape changes mediated by SUN. Yet, how SUN accomplishes changes in cell division patterns is poorly understood. The IQD members share a common central motif of 67 conserved residues named the IQ67 domain that binds calmodulin (CaM) (Abel et al., 2005; Levy et al., 2005; Huang et al., 2013). High expression of the first identified member of the family, *AtIQD1*, leads to increases in glucosinolates (Levy et al., 2005), a class of secondary metabolites involved in plant defense that is absent from Solanaceous plants. How increases in glucosinolate levels in Arabidopsis relate to fruit shape changes in tomato is therefore, not clear. High expression of *SUN* leads to phenotypes associated with auxin homeostasis, yet direct links with auxin through signaling and hormone levels have not been established (Wu et al., 2011; Clevenger, 2012). A recent Y2H study demonstrated that Arabidopsis IQD1 interacts with CaM/CMLs and kinesin light chain-related protein-1 (KLCR1), the latter acts as a motor for transport of vesicles, organelles, mRNA-protein complexes within the cytoplasm along microtubules (Burstenbinder et al., 2013). The directional transport of cargo by kinesins could involve the regulation of cell division patterns (Hirokawa et al., 2009; Akhmanova and Hammer, 2010; Verhey et al., 2011). The association of AtIQD1 with microtubules suggests that it acts as a scaffold protein to recruit cargo to kinesin motors for directional transport along microtubules (Burstenbinder et al., 2013). Whether SUN plays a similar role as AtIQD1 by interaction with KLCR1 proteins is unknown. However, the possible involvement in transport of cargo and the regulation of cell division patterns would suggest that the mutant version of *SUN* that is highly expressed in developing flowers may act as early as stage 3 in organ development, similarly to *OVATE* (Figures 2B,C, Table 1).

### CNR/FW2.2

The first fruit weight QTL that was cloned from vegetables and fruit crops was *FW2.2* (Frary et al., 2000). The locus was fine mapped to the bottom of chromosome 2 and found to encode a member of a novel family of cysteine-rich proteins that share the PLAC8 motif (Guo et al., 2010). The family is known to regulate cell number, hence the new name for *FW2.2-like* genes: *Cell Number Regulator* (*CNR*) (Guo et al., 2010; Guo and Simmons, 2011) (Figure 3). The underlying mutation to cause changes in fruit weight was predicted to be in the promoter as there were no polymorphisms in the coding region of the gene (Frary et al., 2000). Association mapping led to the identification of a putative promoter mutation that underlies the fruit weight changes (Figure 3). Expression of *CNR/FW2.2* is in general low, except in the root, young flower buds and developing fruit (Figure 4A). Its expression is also the highest in the IM/FM reducing to nearly undetectable levels in the floral buds 6 days after initiation (Figure 4B). The allele increasing fruit weight causes the enlargement of the placenta and columella regions of the fruit (Cong et al., 2002; Gonzalo et al., 2009). Previous studies suggested that the members of the CNR family are localized to the membrane facilitating the transport of ions such as cadmium (Song et al., 2004) and calcium (Nakagawa et al., 2007) across membranes (Guo et al., 2010; Libault et al., 2010). Very little additional information is known about the function of CNR/FW2.2 and how regulation of ion transport would lead to changes in cell division. Ovary size is different at anthesis, implying that CNR/FW2.2 acts early during development of the gynoecium. Based on expression profile, the promoter mutation may result in fruit weight changes as early as phase 1 or 2 (Table 1).

## Tomato fruit weight and shape genes acting post-anthesis

### SUN

*SUN* clearly impacts the patterning of the fruit prior to anthesis (see above). However, the most dramatic effect of *SUN* on shape is manifested after anthesis, during phase 5, which is the cell division stage of fruit development (Van Der Knaap and Tanksley, 2001; Xiao et al., 2009) (Figure 6B). As a result of *SUN* expression, cell number was much higher along the proximal-distal axis and lower along the medio-lateral axis at 7 days post anthesis compared to anthesis (Wu et al., 2011) which are likely due to the changes in cell division rates in one direction over another and not the duration of cell division since fruit ripening time is not altered (data not shown). The proposed changes in cell division rates in different tissues of the developing fruit is likely because fruit weight is not altered and thus SUN appears to result in a redistribution of mass. This change in shape is accompanied by changes in gene expression profiles that are specific to the developing pericarp and columella, especially for genes related to cell division (Clevenger, 2012). These findings suggest that the differences in growth along the various axes after anthesis are accompanied by differential gene expression to achieve the final fruit shape. These differences in gene expression in the different tissue types cede at the time when fruit shape mediated by *SUN* is final which is around 10 days post-anthesis (Clevenger, 2012).

### SLKLUH/FW3.2

The second fruit weight QTL identified from vegetable and fruit crops is *FW3.2* (Chakrabarti et al., 2013). The gene was fine mapped to the bottom of chromosome 3 encoding a cytochrome P450 of the CYP78A class and the likely ortholog of Arabidopsis *KLUH* (Zhang et al., 2012; Chakrabarti et al., 2013) (Figure 3). Based on association mapping and additional segregation experiments, a mutation in the promoter of *SlKLUH* is proposed to underlie the change in tomato fruit weight. This mutation is located 512 bp upstream of the predicted start of *SlKLUH* transcription in a putative *cis*-element that is known as an organ-specific element found in nodulin and leghemoglobin genes (Stougaard et al., 1990; Chakrabarti et al., 2013) (Figure 3). Expression of tomato *KLUH* is high in young growing tissues containing meristems or developing seeds (Figure 4A). Also, its expression is particularly high in the IM/FM and decreases in the developing flower buds (Figure 4B). Moreover, within the fruit, *KLUH* is very highly expressed in the developing seeds and much lower in the developing pericarp (Chakrabarti et al., 2013).

The mutant allele of *SlKLUH*, found in many cultivated tomato accessions, does not impact ovary size at anthesis; rather its effect on fruit weight becomes apparent 3 weeks post-anthesis (Zhang, 2012). Yet, transgenic down regulation of *SlKLUH* led to shorter plants and leaves, smaller flowers in addition to reduced fruit weight (Chakrabarti et al., 2013). This result implies that the role of KLUH in plant development is broader than the differences in the function of the natural KLUH alleles demonstrate. The increase in fruit weight arises primarily from increased pericarp and septum areas, resulting from additional number of cells. The increases in cell number is likely the result of a change in duration of cell division and not the rate since fruit ripening is delayed as well. In addition to fruit weight, *SlKLUH* has a pleiotropic effect on branching behavior. The large fruit allele of *SlKLUH* causes reduced branch number and length as well as fewer fruits. This leads to comparable yields from NIL plants carrying the wild type or the mutant *SlKLUH* allele (Chakrabarti et al., 2013).

It has been hypothesized that KLUH generates a mobile growth promoting signal different from the known phytohormones. However, the exact molecular and biochemical nature of the “mobile” signal remains elusive and the substrate for this subfamily of P450 enzymes is also yet to be deciphered (Anastasiou et al., 2007; Adamski et al., 2009).

## Do orthologs of tomato fruit weight and shape genes impact fruit morphology in other domesticated plants?

The domestication of fruit and vegetable crops was likely driven by selections for increases in fruit weight and shape in many incipient crop species. Thus, the question arises whether any of the tomato genes or members of their families are associated with fruit weight and shape in other species. Of the fruit weight genes, other members of the CYP78A class to which *SlKLUH/FW3.2* belongs are known to regulate floral organ and fruit size, leaf and seed size, embryo and endosperm size, apical dominance and plastochron length in Arabidopsis, moss and rice (Ito and Meyerowitz, 2000; Miyoshi et al., 2004; Anastasiou et al., 2007; Adamski et al., 2009; Katsumata et al., 2011; Fang et al., 2012; Nagasawa et al., 2013). More intriguingly, in *Capsicum spp* (chile pepper), *Cucumis melo* (melon) and *Vitis vinifera* (grape), the putative ortholog of *KLUH* and members of the same CYP78A class were associated with larger fruit, suggesting a possible role of this small and largely unknown cytochrome P450 family in parallel domestication processes in fruit and vegetable crops (Chakrabarti et al., 2013; Doligez et al., 2013; Monforte et al., 2014). Collectively, these findings point toward an evolutionarily highly conserved function for this subfamily of P450s in regulating plant organ size. For *CNR/FW2.2*, members of the family regulate plant growth and biomass as well as ear length and kernel number per row in maize (Guo et al., 2010) and the number of nitrogen-fixing nodules in soybean (Libault et al., 2010). QTL studies into the regulation of fruit weight in chile pepper, melon and cherry have also implied a possible role for *FW2.2/CNR-like* genes to control weight in a range of crop species (Paran and Van Der Knaap, 2007; De Franceschi et al., 2013; Monforte et al., 2014).

Of the fruit elongation genes, down regulation of a member of the OFP family in pepper led to a longer shaped fruit (Tsaballa et al., 2011), whereas in melon several OFP members mapped to fruit shape QTLs (Monforte et al., 2014). This suggests that the OFP family is likely to control shape of other fruit and vegetables. Of the locule number genes, a weakly overexpressed *WUSCHEL*-*like* gene in soybean showed an enlarged gynoecium (Wong et al., 2011) which also implies that natural alleles of *WUS* could impact the size of fruits and vegetables in other crops.

## Conclusions

Recent discoveries have started to shed light on the regulation of fruit shape and weight, and the molecular mechanisms underlying this diversity found in cultivated germplasms. However, these six genes are unlikely to represent the entire repertoire of genes acted on by domestication and diversification. The identification of suppressors of *ovate* (Rodriguez et al., 2013) and the effects of genetic background on the severity of the *lc* and *fas* mutants both provide evidence for the existence of other genes that interact with these major regulators of fruit shape and size. In addition, the identification of additional fruit weight QTLs (Huang and Van Der Knaap, 2011) will result in the identification of new regulators in fruit weight. Further, the exploitation of TILLING mutants that impact shape and weight may also significantly augment the resources available in the fruit morphology tool kit (Okabe et al., 2011). The molecular and biochemical characterization of the genes and encoded proteins in the future will greatly add to our understanding into the pathways regulating the final dimensions of the fruit.

Advancing the research into the function of fruit morphology proteins is going to lead to fundamental insights into plant developmental processes. Especially processes that regulate cell proliferation and enlargement patterns, as well as its rate and duration are of particular importance since they pertain to growth of all plant organs and eventually yield. In all, the discoveries made using tomato fruit morphology as a model will undoubtedly support fundamental and applied research that is applicable to many other plant systems.

## Author contributions

All authors contributed critically to the writing and editing of the manuscript, agree to be accountable for the data presented and approve the version of the manuscript. Esther van der Knaap wrote the manuscript and constructed Figure 3, Table 1. Manohar Chakrabarti contributed the section about KLUH. Yi Hsuan Chu and Zejun Huang contributed to the section about LC and FAS and Figures 1, 5. Josh P. Clevenger, Liang Sun, and Yanping Wang contributed to the section about SUN. Eudald Illa-Berenguer and Qi Mu contributed to the section about CNR, Table 2, and Figure 4. Neda Keyhaninejad and Shan Wu contributed to Figure 7 and Table 3. Shan Wu contributed to the section about OVATE and Figures 2, 6.

### Conflict of interest statement

The authors declare that the research was conducted in the absence of any commercial or financial relationships that could be construed as a potential conflict of interest.
